# Enhancing Diagnosis of Autism With Optimized Machine Learning Models and Personal Characteristic Data

**DOI:** 10.3389/fncom.2019.00009

**Published:** 2019-02-15

**Authors:** Milan N. Parikh, Hailong Li, Lili He

**Affiliations:** ^1^Perinatal Institute, Cincinnati Children’s Hospital Medical Center, Cincinnati, OH, United States; ^2^Department of Pediatrics, University of Cincinnati College of Medicine, Cincinnati, OH, United States

**Keywords:** autism spectrum disorder, machine learning, diagnosis, biostatistics, support vector machine

## Abstract

Autism spectrum disorder (ASD) is a developmental disorder, affecting about 1% of the global population. Currently, the only clinical method for diagnosing ASD are standardized ASD tests which require prolonged diagnostic time and increased medical costs. Our objective was to explore the predictive power of personal characteristic data (PCD) from a large well-characterized dataset to improve upon prior diagnostic models of ASD. We extracted six personal characteristics (age, sex, handedness, and three individual measures of IQ) from 851 subjects in the Autism Brain Imaging Data Exchange (ABIDE) database. ABIDE is an international collaborative project that collected data from a large number of ASD patients and typical non-ASD controls from 17 research and clinical institutes. We employed this publicly available database to test nine supervised machine learning models. We implemented a cross-validation strategy to train and test those machine learning models for classification between typical non-ASD controls and ASD patients. We assessed classification performance using accuracy, sensitivity, specificity, and area under the receiver operating characteristic curve (AUC). Of the nine models we tested using six personal characteristics, the neural network model performed the best with a mean AUC (SD) of 0.646 (0.005), followed by k-nearest neighbor with a mean AUC (SD) of 0.641 (0.004). This study established an optimal ASD classification performance with PCD as features. With additional discriminative features (e.g., neuroimaging), machine learning models may ultimately enable automated clinical diagnosis of autism.

## Introduction

Autism spectrum disorder (ASD) is characterized by impaired linguistic, communication, cognitive and social skills (Wetherby and Prutting, 1984). Therapies have been developed to treat the varying degrees of symptoms and improve patient quality of life. However, the diagnosis of ASD remains challenging, especially for marginal cases, resulting in under- and over-diagnosis. To date, behavior-based tests are the standard clinical approach to diagnosing ASD (American Psychiatric Association, 2013). The diagnostic process for ASD is time-consuming and costly (Galliver et al., 2017). An automated ASD diagnostic approach might allow for earlier identification of ASD and could help provide a map of high-risk populations. Emerging machine learning approaches are showing great promise for objective evaluation of neuropsychiatric disorders (Nielsen et al., 2013; Bone et al., 2015; Chen et al., 2015; Plitt et al., 2015; Ghiassian et al., 2016; Yahata et al., 2016; Abraham et al., 2017).

Machine learning is a group of statistical techniques that learn with the distribution of data so as to make decisions on new data. It is used to devise complex applications to make accurate classifications/predictions on diverse data (Russell and Norvig, 2010). Autism diagnosis could be formulated as a typical classification problem (i.e., ASD vs. typical control/non-ASD). The constructed model/classifier is then able to evaluate whether a new unknown subject has ASD or not based on input features.

Several studies have employed machine learning to improve ASD diagnosis. Duda et al. (2016) applied machine learning to distinguish autism from attention deficit hyperactivity disorder using a 65-item Social Responsiveness Scale. Bone et al. (2015) trained their models to diagnose autism against healthy controls using the same Social Responsiveness Scale and the Autism Diagnostic Interview-Revised scores. More recently, the Autism Brain Imaging Data Exchange (ABIDE) has gathered data [i.e., personal characteristic data (PCD), structural MRI, functional MRI] from over 1,000 subjects and made it available for the ASD research community (Craddock et al., 2013). This has facilitated the development of machine learning models towards the automated diagnosis of ASD (Ghiassian et al., 2016; Abraham et al., 2017; Heinsfeld et al., 2018; Li et al., 2018). While most studies have focused on brain neuroimaging data, few studies have reported automated machine learning models that solely rely on PCD as input features. As such, the full potential of PCD on ASD classification has yet to be comprehensively evaluated. It is important to note that a true diagnostic classifier of ASD cannot be created due to the retrospective case-control ABIDE study design. In this work, we simply set out to assess the predictive power of PCD for ASD diagnosis and evaluate which machine learning model is most robust for this task. Specifically, we employed and validated nine machine learning models by using PCD, such as age, sex, handedness, and IQ, for ASD classification of individual subjects. Taking advantage of such a large PCD dataset from ABIDE, we systematically evaluated the predictive power of PCD features on ASD classification and compared the performance of those nine machine learning models.

## Materials and Methods

### Data

We selected six PCD features of interest—age at testing, sex, handedness, full-scale IQ, verbal IQ, performance IQ—from the ABIDE I Preprocessed Database. Only subjects with information for all 6 features were included (*N* = 851 of total of 1,112 subjects in ABIDE I database). Of the 851 subjects, 430 were typical non-ASD controls and 421 had a confirmed diagnosis of ASD. To control for site effects, we included site of testing in each of the models. Using a two-sided Student’s *t*-test (unequal variance), we identified significant differences between ASD patients and healthy controls in full-scale IQ (*p* < 0.001), verbal IQ (*p* < 0.001), and performance IQ (*p* = 0.003); there was no significant group difference in age (*p* = 0.8582). Sex (*p* = 0.017) and handedness (*p* = 0.018) were also significantly different between groups (chi-squared test; Table 1).

**Table 1:** Demographic information for our sub-sample of the Autism Brain Imaging Data Exchange (ABIDE) Database.

Group	ASD (*N* = 421)	Control (*N* = 430)	*P*
Age	16.8 ± 7.7	16.7 ± 6.9	0.858
Full-Scale IQ	105.2 ± 16.8	110.9 ± 12.6	<0.001
Verbal IQ	104.4 ± 17.8	111.3 ± 13.3	<0.001
Performance IQ	105.0 ± 17.2	108.2 ± 13.3	0.003
Sex (%)			0.017
Male	88	82	
Female	12	18	
Handedness (%)			0.018
Left	13	6	
Right	85	92	
Ambidextrous	2	1	

*All data are mean ± SD unless otherwise specified*.

A portion of the ABIDE study sites defined handedness as a score based on the Edinburgh Handedness Inventory while others coded it as a category (left, right, or ambidextrous). Thus, we reformatted all handedness data to categorical values. This study included 15 different ABIDE recruitment sites. These were included in the features to control for site of testing.

### Classification Models

In order to comprehensively evaluate the full potential of PCD for ASD classification, we tested a variety of approaches, including k-nearest neighbor (Altman, 1992), linear and nonlinear Support Vector Machine (SVM; Cortes and Vapnik, 1995), decision tree (Breiman et al., 1984), logistic regression (Dobson, 1990), Stacked Sparse Auto-encoder (SSAE)-based neural network (Hinton and Salakhutdinov, 2006), random forest (Breiman, 2001), and majority voting and weighted average ensemble models (Cruz and Wishart, 2006; Zhou, 2012). The models are detailed in the Supplementary Materials.

To optimize the performance of each model, we performed a parameters grid search (Cuingnet et al., 2011) for each model (Supplementary Table S1; Supplementary Materials).

### Model Evaluation

We applied a k-fold cross-validation scheme to train and test the models. The whole dataset was randomly divided into 25 equal sized portions. Of the 25 portions, one portion of data was held out for model testing, and the remaining 24 portions were used for model training. In order to create a validation dataset for model optimization, a 10-fold cross-validation was performed on the training dataset for each model (Supplementary Materials; Supplementary Figure S1). This process was repeated until each of the 25 portions was evaluated once as the testing data. We evaluated the model based on the concatenated test labels and ground truth labels across 25 iterations. We repeated this k-fold cross-validation 30 times.

The performance of the classification was assessed using four diagnostic metrics: accuracy, sensitivity, specificity and area under the receiver operating characteristic curve (AUC). Accuracy is measured as the percentage of correctly classified subjects within all subjects. Sensitivity is defined as the percentage of correctly classified ASD subjects within all ASD subjects, while specificity is represented by the percentage of correctly classified healthy subjects within all typical non-ASD control subjects. Sensitivity is the ability of the classifier to correctly identify ASD subjects (true positive rate), whereas specificity is the ability of the classifier to correctly identify healthy subjects (true negative rate). AUC reflects the diagnostic ability of a binary classifier system when its discrimination cutoff varies.

## Results

From the models we tested using all six PCD features, we found that the model with the best AUC was the Stacked Sparse Auto-encoder (SSAE)-based neural network (*p* < 0.001) which correctly classified ASD patients with a mean (SD) accuracy of 62.0% (0.9%) and AUC of 0.646 (0.005; Table 2). The k-nearest neighbor model displayed an accuracy of 61.8% (0.8%) and the second highest AUC of 0.641 (0.004), but its sensitivity was lower than most models. Compared to this, both linear and non-linear SVM yielded better performance considering overall diagnostic measures.

**Table 2:** Accuracy, sensitivity, specificity, and area under the receiver operating characteristic curve (AUC) values for each machine learning model.

	Accuracy (%)	Sensitivity (%)	Specificity (%)	AUC
Decision tree	54.7 ± 1.5	53.3 ± 2.0	54.9 ± 1.7	0.562 ± 0.015
Majority model	61.9 ± 0.8	55.4 ± 1.1	69.2 ± 1.3	0.568 ± 0.009
Random forest	57.2 ± 0.8	54.4 ± 1.2	60.4 ± 1.1	0.615 ± 0.007
SVM (linear)	61.4 ± 0.5	57.1 ± 0.6	66.7 ± 0.8	0.622 ± 0.002
SVM (non-linear)	61.9 ± 0.4	52.3 ± 1.5	71.6 ± 1.1	0.623 ± 0.005
Confidence model	61.5 ± 0.9	49.1 ± 1.4	67.1 ± 1.0	0.633 ± 0.008
Logistic regression	59.1 ± 0.5	55.5 ± 0.6	62.6 ± 0.8	0.635 ± 0.001
k-Nearest neighbor	61.8 ± 0.6	46.6 ± 1.0	72.1 ± 0.8	0.641 ± 0.004
Neural network	62.0 ± 0.9	53.3 ± 1.3	71.2 ± 1.9	0.646 ± 0.005

*All data are mean ± SD; SVM, Support Vector Machine*.

Using a feature selection method based on the Student’s *t*-test, we noted that the most predictive features were full-scale IQ, followed by verbal IQ and performance IQ. By using only these three features, the neural network achieved an AUC (SD) of 0.641 (0.009) which was very comparable to the AUC using all seven features. By removing females (*n* = 126) and only considering male subjects (*n* = 725), the diagnostic performance for neural network was also comparable with an accuracy of 61.1% (1.3%) and AUC of 0.645 (0.014).

## Discussion

This study set out to explore the full potential of PCD as diagnostic features for ASD classification. We developed and compared nine automated machine learning models by using a large PCD dataset from the ABIDE repository. In our evaluation, our neural network model outperformed eight other peer models by achieving the best AUC of 0.646.

PCD have demonstrated strong predictive power for other neurodevelopmental disorders. For example, in the ADHD-200 global competition, PCD features outperformed fMRI features in attention deficit hyperactivity disorder classification (Brown et al., 2012). This inspired us to test the predictive power of PCD for ASD classification. Previous studies using PCD for ASD classification have been limited, and optimal performance for PCD has not been established. In recent studies, PCD were only investigated for the purpose of feature fusion or integration. For instance, Ghiassian et al. (2016) reported an accuracy of 59.6% with non-linear SVM using the same six PCD features and eye stat (eyes open or closed). However, they investigated PCD performance only for model comparison. In addition, their results were based on one classifier whereas we tested multiple classifiers to determine not only the best performance but also the model that consistently yielded the best performance. Finally, when we used the same dataset as Ghiassian et al. (2016) in our neural network model, we obtained a somewhat higher accuracy of 62.3%. Nevertheless, these differences might have also resulted from other factors such as study differences in cross-validation. The more important takeaway is that the six PCD we tested, and particularly the three IQ measures, provide significant predictive power for ASD diagnosis that should be incorporated into future ASD classification studies.

Our results highlight the advantage of neural networks over other commonly employed machine learning models in ASD classification. Traditionally, neural network models have had a significantly higher computational cost than other peer models. With recent rapid advances in deep learning techniques, the current techniques have reduced the optimization process for neural networks to an acceptable training time. As shown in Supplementary Table S1, the neural network model has more hyperparameters which provide the model with additional flexibility to learn the PCD distribution for ASD classification. Interestingly, k-nearest neighbor had the second-best AUC among our nine models, but its sensitivity in our experiment was not desirable. Compared to this, both linear and non-linear SVM yielded better performance considering overall diagnostic measures.

In addition, our results compare favorably to recent predictions made using fMRI features from a similar sample of the ABIDE database (Abraham et al., 2017). That study achieved a maximum accuracy (SD) of 66.8% (5.4%). Although our model with PCD had a lower accuracy of 62.0%, our standard deviation of 0.9% is substantially lower (i.e., narrower confidence interval) than their model. Additionally, our model only requires six simple PCD features which are low-cost and easy-to-obtain as compared to neuroimaging data. These performance scores compared to fMRI-based classification emphasize the importance of PCD in ASD classification.

The main limitations of our study arise from how the ABIDE data were collected. This international study collected data from 17 unique clinical and research sites. This leads to heterogeneity in the data that might compromise the machine learning models. To mitigate the impact of site bias, we controlled for the site of testing by including it in all the models. However, the heterogeneity of PCD data may require further investigation before such models can be utilized in clinical settings. The small sex difference in ASD vs. controls we observed is likely a function of the high incidence of ASD in males rather than a selection bias for this substudy. Even if this was a biased selection from ABIDE, our secondary analyses in only males from this subpopulation yielded very similar results to our primary analysis that included both sexes, suggesting this difference did not affect performance or bias our results. Another limitation is the size of the dataset. While 851 subjects are considered a large study in this field of clinical research, larger datasets may be needed to yield generalizable machine learning models. Also, our ASD classifiers specifically focused on the classification of ASD and would not be effective in detecting the presence of other developmental disorders. A large prospective study of a more heterogeneous population would be required to confirm the value of PCD and/or other promising features to diagnose ASD.

Future efforts could include combining PCD with neuroimaging data using machine learning models. Along with the addition of fMRI features, the use of other features, such as medical tests or past or family history of disease, might boost the performance of the models to a clinically useful level. The addition of more features may also increase the performance of neural networks and allow for the use of more complex architecture of neural networks. Studies testing new machine learning models show promising results using fMRI features (He et al., 2018; Li et al., 2018). A recent development in machine learning, called transfer learning, mimics the human brain by using large amounts of available information unrelated to the disease of interest (e.g., typical controls) to draw conclusions when presented with a smaller, less accessible amount of information about the disease of interest. Transfer learning has already been shown to improve classification and identify networks in the brains of high-risk premature birth babies (He et al., 2018) and diagnose autism on small subsets of the ABIDE database (Li et al., 2018).

In summary, we developed and compared nine machine learning models for ASD classification by using PCD as input features. We conclude that combining PCD with optimized machine learning models can enhance diagnosis of ASD. When integrated with additional features (e.g., fMRI features), these models have the potential to yield a more objective approach for diagnosing autism.

## Data Availability

The dataset analyzed for this study was the international ABIDE dataset, which can be accessed here: www.preprocessed-connectomes-project.org/abide/index.html. All generated data for this study are included in the manuscript.

## Author Contributions

LH and HL conceived the project. MP organized the experiments and analyzed the data with guidance from HL. MP wrote the first draft of the manuscript. All authors contributed to the manuscript revision, read and approved the submitted version.

## Conflict of Interest Statement

The authors declare that the research was conducted in the absence of any commercial or financial relationships that could be construed as a potential conflict of interest.

## Abbreviations

ABIDE: Autism Brain Imaging Data Exchange
ASD: Autism Spectrum Disorder
AUC: Area Under the Receiver Operating Characteristic Curve
PCD: Personal Characteristic Data
SVM: Support Vector Machine.
