# Supplementary material for: Enhancing Diagnosis of Autism With Optimized Machine Learning Models and Personal Characteristic Data
Source: Front Comput Neurosci. 2019 Feb 15;13:9. doi: 10.3389/fncom.2019.00009 (PMC6384273; doi:10.3389/fncom.2019.00009)

**Enhancing Diagnosis of Autism With Optimized Machine Learning Models and Personal Characteristic Data**

*Milan N. Parikh 1 , Hailong Li 1 and Lili He 1,2*

*1Perinatal Institute, Cincinnati Children’s Hospital Medical Center, Cincinnati, OH, United States, 2Department of Pediatrics,* *University of Cincinnati College of Medicine, Cincinnati, OH, United States*

**Supplementary Material**

**Machine learning models**

***K-nearest neighbor:***

In the k-nearest neighbor model, we applied Euclidean distance as the distance measurement between the PCD features of the samples (i.e., subjects). The number of nearest neighbors was optimized from 10 to 30.

***Linear and nonlinear Support Vector Machine (SVM):***

Each PCD feature was normalized into a [0, 1] scale to prevent any feature from dominating the optimization of SVM models. For the linear SVM model, we searched the hyperparameter margin penalty with empirical values [2^-2^, 2^-1^, …, 2^7^, 2^8^]. For nonlinear SVM, we chose the Radial Basis Function (RBF) kernel. By using a grid search method, we searched the margin penalty [2^-2^, 2^-1^, …, 2^7^, 2^8^], and kernel scale gamma [0.5, 1.0, 1.5, 2.0, 2.5]

***Decision tree:***

For the decision tree model, we applied the CART algorithm (Breiman, L., et al. Classification and Regression Trees, 1984) to create decision trees. This starts with all 6 PCD features and select a split with the best optimization criterion. To avoid a split leading to a child node having too few observations, we also applied a 20% as a constraint. Then, this process is repeated recursively for the two child nodes.

***Random forest:***

For the random forest model, we optimized the number of trees by searching the empirical values [50, 60, 70, 80, 90, 100]. The number of trees was selected when the AUC was the best on the training data.

***Logistic regression:***

Maximum-likelihood estimation algorithm was utilized to optimize the coefficient of logistic regression model. We also normalized the scale of 6 PCD feature into [0, 1] to prevent the large scale feature, such as IQ, to dominate the optimization process of model.

***Neural networks:***

In terms of the neural network model, we implemented the Stacked Sparse Auto-Encoder (SSAE) as the method to build a feedforward neural network. According to the small feature set, we designed a 3-layer neural network with an input layer, one hidden layer and an output layer. The number of nodes in the hidden layer was searched among [2, 3, 4, 5]. The building process includes pre-training, supervised learning, and fine tuning steps. A loss function with L2 regularization and sparsity regularization terms was adopted from (Olshausen, B. A. and D. J. Field. “Sparse Coding with an Overcomplete Basis Set: A Strategy Employed by V1.” Vision Research, Vol.37, 1997, pp.3311–3325.). During model optimization, we tested sparsity proportion [0.1, 0.2, 0.3, 0.4, 0.5] and L2 regularization weight [0.1, 0.2, 0.3, 0.4, 0.5]

***Majority voting ensemble model*.**

The majority voting technique considers the predictions by each above non-ensemble model as a ‘vote,’ and the predictions derived from the majority of the models are used as the final prediction (Zhou, 2012. Ensemble Methods: Foundations and Algorithms). We calculated area under the receiver operating characteristic curve (AUC) with the posterior probabilities among the models that had predicted the majority label for each sample.

***Weighted average ensemble models***

The weighted average technique calculates a weighted average of predictions from all the above non-ensemble models and uses it to make the final prediction. The assigned different weights define the importance of each model for prediction (Zhou, 2012. Ensemble Methods: Foundations and Algorithms).

Optimized hyperparameters of individual models are summarized in Supplementary Table 1**.**

| Model | Parameter | Optimization |
| --- | --- | --- |
| kNN | Number of Neighbors | $10 :30$ |
| Linear SVM | Margin Penalty | $2^{-2 :8}$ |
| SVM-RBF | Margin Penalty | $2^{-2 :8}$ |
|  | Kernel Scale | $0.5 :0.5 :2.5$ |
| Neural Network | Sparsity Proportion | $0.1 :0.1 :0.5$ |
|  | L2 Weight Regularization | $0.1 :0.1 :0.5$ |
| Random Forest | Number of Decision Trees | $50 :10 :100$ |
| Supplementary Table 1. The ranges of values used by each model type for parameter optimization. Ranges are formatted as start:step:stop where step is assumed to be 1. | | |

**Model Validation Scheme**

We applied a nested k-fold cross-validation scheme to evaluate the models. Briefly, this scheme includes an outer loop and an inner loop. (Supplementary Figure 1) In the outer loop, the whole data set was split into training and testing dataset. Inside the outer loop, another round of cross-validation (e.g., ten-fold cross-validation) was performed in an inner loop on the input training dataset to create both training and validation datasets. The models used by this study were executed as subfunctions. These subfunctions took inputs of features and classes for training the model as well as features for testing the model. The outputs were the predicted classes as well as the scores with which the model created the binary classification. Each model had parameters to be optimized and ranges of values for those parameters selected (Supplementary Table 1). The model was trained and optimized in the inner loop. The model producing the highest AUC was used to test the testing data in the outer loop.  In the outer loop, the training and optimization processes were only conducted on the training data, and the testing data were used as a blind test.


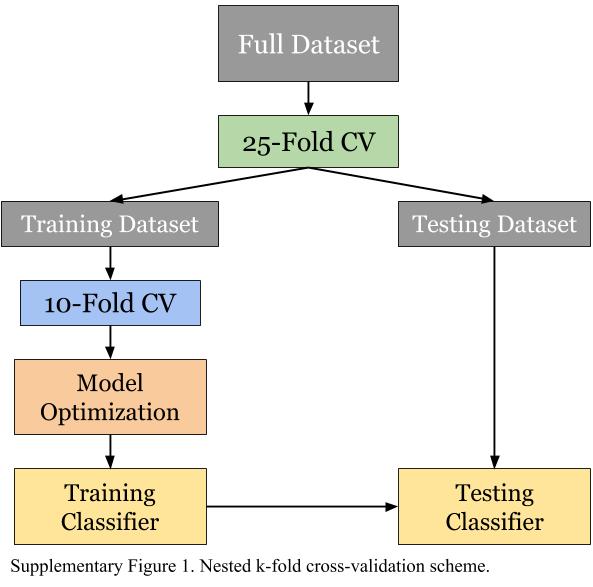

Supplement: Supplementary file 1 [file Data_Sheet_1.docx]
